# Screening of disease-related biomarkers related to neuropathic pain (NP) after spinal cord injury (SCI)

**DOI:** 10.1186/s40246-021-00303-w

**Published:** 2021-01-25

**Authors:** Jia Zhao, Li Yang, Limin Huang, Zinan Li

**Affiliations:** 1 Department of Internal Neurology, The Third Hospital of Jilin University, 126 Xiantai Street, Changchun, 130000 Jilin, People’s Republic of China; 2 Department of Internal Neurology , The Third Hospital of Jilin University , 126 Xiantai Street, Changchun, 130000 Jilin People’s Republic of China

**Keywords:** Spinal cord injury (SCI), Neuropathic pain (NP), Disease-related biomarkers

## Abstract

**Background:**

Based on the molecular expression level, this paper compares lncRNA and mRNA expressions respectively in peripheral blood samples of the patients after SCI with NP and without NP, and screens disease-related biomarkers related to NP after SCI in peripheral blood samples of patients.

**Method:**

The expression spectrum of 25 human peripheral blood samples (12 samples of refractory NP patients after SCI) was downloaded and data were normalized. Screening of GO annotations significantly associated with significant differentially expressed mRNAs and significant involvement of the KEGG pathway. The WGCNA algorithm was used to screen for modules and RNAs that were significantly associated with disease characterization. A co-expression network was constructed to extract the genes involved in the disease pathway from the co-expression network, construct a network of SCI pain-related pathways, and screen important disease-related biomarkers. Quantitative real-time PCR was used to detect the mRNA expression of hub genes.

**Results:**

Data were normalized and re-annotated by detection of platform information, resulting in a total of 289 lncRNA and 18197 mRNAs. Screening resulted in 338 significant differentially expressed RNAs that met the threshold requirements. Differentially expressed RNAs were significantly enriched with the brown and magenta modules. Six KEGG signaling pathways were screened in the co-expression network, and three KEGG pathways with direct neuropathic pain were identified. The expression levels of E2F1, MAX, MITF, CTNNA1, and ADORA2B in the disease group were all significantly upregulated (*p* < 0.01). Compared with the normal group, the expression of OXTR was upregulated.

**Conclusion:**

We speculate that there are 7 genes and 2 lncRNAs directly involved in the pain pathway: E2F1, MAX, MITF, CTNNA1, ADORA2B, GRIK3, OXTR, LINC01119, and LINC02447. These molecules may be important for NP after SCI.

**Supplementary Information:**

The online version contains supplementary material available at 10.1186/s40246-021-00303-w.

## Introduction

Spinal cord injury (SCI) refers to the damage of the spinal cord due to various pathogenic factors and is a common clinical disease in orthopedics [1]. SCI is a multi-stage, multi-pathway, and multi-factorial pathological process that often leads to severe neurological dysfunction [2]. This includes primary injuries in the early stages of spinal cord injury and consequent secondary ischemia, edema, and secondary damage caused by reperfusion after ischemia. Due to the non-renewable characteristics of nerves, especially the central nervous system, and high disability rate, it has been plaguing the majority of patients, bringing psychological and economic burden [3]. Studies have shown that primary damage caused by the early stage of spinal cord injury leads to the loss of a large number of nerve cells, and the subsequent secondary damage causes neuronal apoptosis, loss of nerve cells, and non-renewable, and keratinocyte regeneration. The formation of nerve scars also hinders the growth of nerve fibers, and exogenous factors hinder regeneration after spinal cord injury [4]. However, in recent years, a large number of studies have shown that endogenous factors may have a greater effect on nerve repair after spinal cord injury [5].

SCI often causes neuropathic pain (NP). Patients with NP are not only unbearable, but long-term pain affects their sleep, work, and life [6]. Research on this field is currently based on animal models, but research reports on human whole blood are rare.

Previous studies have shown that changes in different levels of gene expression will eventually lead to dysregulation of gene expression. Among these regulatory factors, non-coding RNA is getting more and more attention from scholars. Non-coding RNAs are a class of genomic transcription products that have no protein-coding function. More and more studies have confirmed that non-coding RNAs, although not translated into proteins, are involved in all levels of gene expression [7].

Recent studies have shown that long noncoding RNAs are a class of ncRNAs that are greater than 200 nt in length and lack the ability to encode proteins. Compared to RNA encoding proteins, lncRNAs are shorter in length, fewer exons, less coding, and are tissue or cell specific [8].

Spinal cord injury is a complex pathological process involving a large number of cellular and molecular changes. In recent years, many studies have found that lncRNA is enriched in the central nervous system of mice and affects the development of nerves, and is closely related to many nervous system diseases. LncRNA can regulate the expression of coding genes at different levels, and the mechanism is complex [9]. Therefore, studying the differential expression of lncRNA in spinal cord injury will help us further clarify the pathological process of spinal cord injury. At present, more and more studies on the function of lncRNA have found that its function is complex, and it can participate in the regulation of various stages of gene expression, and exert its effects by affecting the molecular level. Numerous evidences suggest that differential expression of lncRNA is closely related to many human diseases [10]. However, there has not been a study on lncRNA expression spectrum in the pathological process of spinal cord injury. By analyzing the signaling pathways of differential genes, it is helpful to study genes and expressions as a whole network. The greater the enrichment value, the closer the differential gene is involved in the signal pathway [11].

Based on the molecular expression level, this paper compares lncRNA and mRNA expressions respectively in peripheral blood samples of the patients after SCI with NP and without NP, and screens disease-related biomarkers related to NP after SCI in peripheral blood samples of patients.

## Materials and methods

### Grouping and preprocessing of experimental data

We downloaded the data numbered E-GEOD-69901 from the European Bioinformatics Institute (EBI) ArrayExpress database (https://www.ebi.ac.uk/arrayexpress/) [12]. The detection platform is GPL15207 (PrimeView) Affymetrix Human Gene Expression Array. The data included 25 human peripheral blood samples, 12 of which were samples of refractory NP after SCI, and 13 were control samples without NP after SCI (Supplementary Table 1). The original format provided by the database we downloaded is the expression spectrum of CEL. The data in the original CEL format is converted using oligo Version 1.41.1 [13] (http://www.bioconductor.org/packages/release/bioc/html/oligo.html) in the R3.4.1 language. The median method was used to complement the missing values, the background correction (MAS method), and the quantile method were used to normalize the data.

### Screening of significant differentially expressed RNA

Download the detailed comment information of the detection platform GPL15207 corresponding to the E-GEOD-69901 data set (https://www.ncbi.nlm.nih.gov/geo/query/acc.cgi?acc=GPL15207). Based on the information provided by the platform, such as Transcript ID, RefSeq ID, and location, the mRNA and lncRNA in the expression spectrum were re-annotated using 4328 lncRNAs and 19,029 protein-coding genes contained in the HUGO Gene Nomenclature Committee (HGNC) [14] (http://www.genenames.org/) database.

According to the sample information, the sample was divided into 12 SCI-pain and 13 SCI-no_pain CTRL control sample groups. Then, using the Limma Version 3.34.0 [15] (https://bioconductor.org/packages/release/bioc/html/limma.html) in the R3.4.1 language, the differential expression FDR values and the expression fold change values of the genes between the comparison groups were calculated. FDR values < 0.05 and |logFC| > 0.5 were selected as thresholds for screening for significant differentially expressed RNA. Based on the expression level of RNAs obtained by screening, using the pheatmap Version 1.0.8 in R3.4.1 language [16] (https://cran.r-project.org/package=pheatmap), the expression values were hierarchically clustered [17, 18] based on Euclidean bidirectional and displayed by heat map. Subsequently, the mRNA in the significant differentially expressed RNA obtained by screening was subjected to a DAVID 6.8-based [19, 20] (https://david.ncifcrf.gov/) GO node analysis and a significant enrichment analysis of the KEGG signaling pathway. Screening for GO annotations that are significantly associated with significant differentially expressed mRNAs, as well as significant involvement of the KEGG pathway.

### Screening for significantly related modules and RNAs for disease characterization using the WGCNA algorithm

Weighed gene co-expression network analysis (WGCNA) is a typical system biology algorithm for constructing co-expression networks. The algorithm is based on high throughput expression data. Firstly, it is assumed that the constructed network obeys the scale-free network, and defines the co-expressing correlation matrix, the adjacency function formed by the network, and then calculates the different coefficients of different nodes to identify the set modules associated with the disease [21, 22]. Here, we use the WGCNA package in R3.4.1 [23] (https://cran.r-project.org/web/packages/WGCNA/) to analyze all RNAs detected in the data set and to screen the modules and the RNA contained therein that are significantly associated with the disease state. The setup RNA module contains a minimum of 100 RNA elements, cutHeight = 0.99.

The significant differentially expressed RNA screened in 2.2 was then mapped into each of the WGCNA color modules obtained in 2.3. By means of *f*(*k*,*N*,*M*,*n*) = *C*(*k*,*M*) × *C*(*n*−*k*,*N*−*M*)/*C*(*n*,*N*) () hypergeometric algorithm [24], the significant enrichment parameter fold enrichment and enrichment significance *p* value of differential RNA in the module are calculated (where *N* represents all RNA involved in the analysis of the WGCNA algorithm, *M* represents the number of RNAs in each module obtained by the WGCNA algorithm, *n* represents the number of significant differences in the number of RNAs screened in 2.2, and *k* represents the number of significant differentially methylated genes mapped into the corresponding modules). The module screening threshold was chosen to be *p* < 0.05, fold enrichment > 1.

### Construction of co-expression network

The lncRNA and mRNA contained in the target module of the significantly enriched distribution screened in 2.3 were calculated by the cor function (http://77.66.12.57/R-help/cor.test.html) in the R3.4.1 language to calculate their expression level Pearson correlation coefficient (PCC). A co-expression network between the significant differentially expressed RNAs was constructed by expression association. The network is visualized by Cytoscape 3.6.1 [25] (http://www.cytoscape.org/). Thereafter, using DAVID, a significant correlation analysis of the KEGG pathway was performed on genes in the co-expression network.

### Construction of the pathway network related to SCI pain

In the Comparative Toxicogenomics Database 2019 update database (http://ctd.mdibl.org/) [26], the “neuropathic pain” was used as a keyword to search for the KEGG pathway directly related to NP after SCI, and the corresponding pathways in the co-expression network were significantly compared, and the overlapping pathways were obtained. The genes involved in the disease pathway were separately extracted from the co-expression network, a network of SCI pain-related pathway was constructed, and important disease-related biomarkers were screened.

### Quantitative real-time PCR clinical trial verification

A total of 6 whole blood samples including 3 healthy samples and 3 samples of refractory NP patients after SCI were collected from China-Japan Union Hospital of Jilin University (Changchun, Jilin, China). Total RNA was isolated from whole blood samples using RNAiso Plus (Trizol) (TAKARA, 9109) reagent according to the manufacturer’s protocols. Follow the kit instructions for mRNA reverse transcription and fluorescence quantitative PCR amplification (PrimeScript™RT Master Mix (Perfect Real Time), TAKARA, RR036A; PrimeScript™ II 1st Strand cDNA Synthesis Kit, TAKARA, 6210A; Power SYBR Green PCR Master Mix, Thermo, 4367659). The primers were listed in Table 1. For statistical analysis, the graphing software was Graphpad prism 5 (Graphpad Software, San Diego, CA), *p* < 0.05 and *p* < 0.01 were the screening criteria for significant and extremely significant differences.

**Table 1:** List of the primers

Primers	Primer sequence (5′-3′)
GAPDH-hF	TGACAACTTTGGTATCGTGGAAGG
GAPDH-hR	AGGCAGGGATGATGTTCTGGAGAG
E2F1-hF	CATCAGTACCTGGCCGAGAG
E2F1-hR	CCCGGGGATTTCACACCTTT
MITF-hF	TGAGCTTGCCATGTCCAAAC
MITF-hR	ACGCTCGTGAATGTGTGTTC
CTNNA1-hF	CCATGCAGGCAACATAAACTTC
CTNNA1-hR	AGGGTTGTAACCTGTGTAACAAG
ADORA2B-hF	TGCACTGACTTCTACGGCTG
ADORA2B-hR	GGTCCCCGTGACCAAACTT
MAX-hF	GAGAGCGACGAAGAGCAACC
MAX-hR	GCACTTGACCTCGCCTTCT
GRIK3-hF	TTCGAGGCGACCAAAAAGG
GRIK3-hR	GGTTCACGTAGAAGGTGTCCT
OXTR-hF	CTGCTACGGCCTTATCAGCTT
OXTR-hR	CGCTCCACATCTGCACGAA

## Results

### Preprocessing of data and screening of significant differential expression

**Fig. 1:**
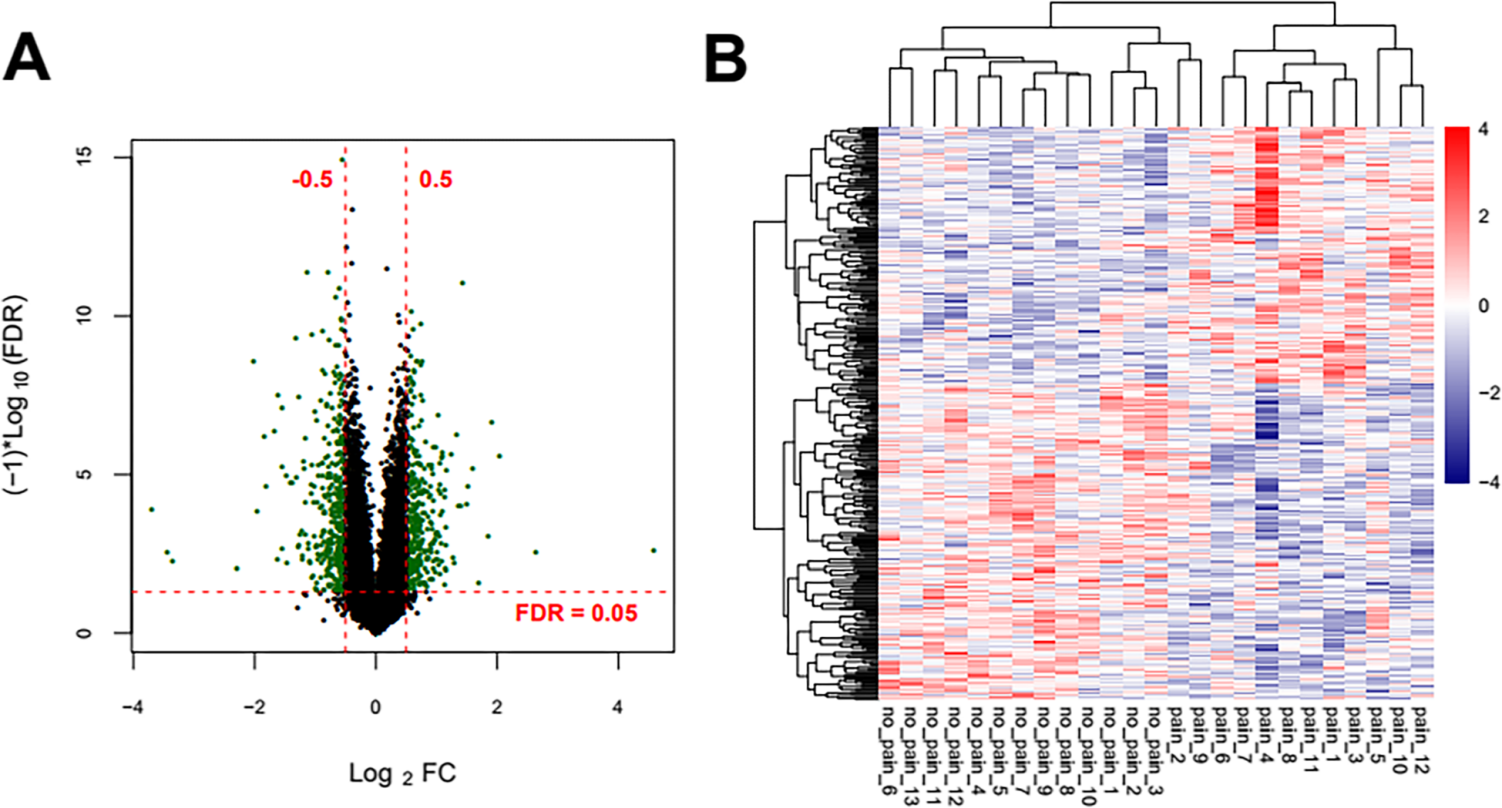
**a** Significant differentially expressed RNA-log_2_FC-log_10_ (FDR) volcano map. Green dots indicate significant differentially expressed RNAs, red horizontal dashed lines indicate FDR < 0.05, and two red vertical lines indicate |Log2FC| > 0.5. **b** Bidirectional hierarchical clustering heat map based on expression levels of the significant differentially expressed RNAs

**Fig. 2:**
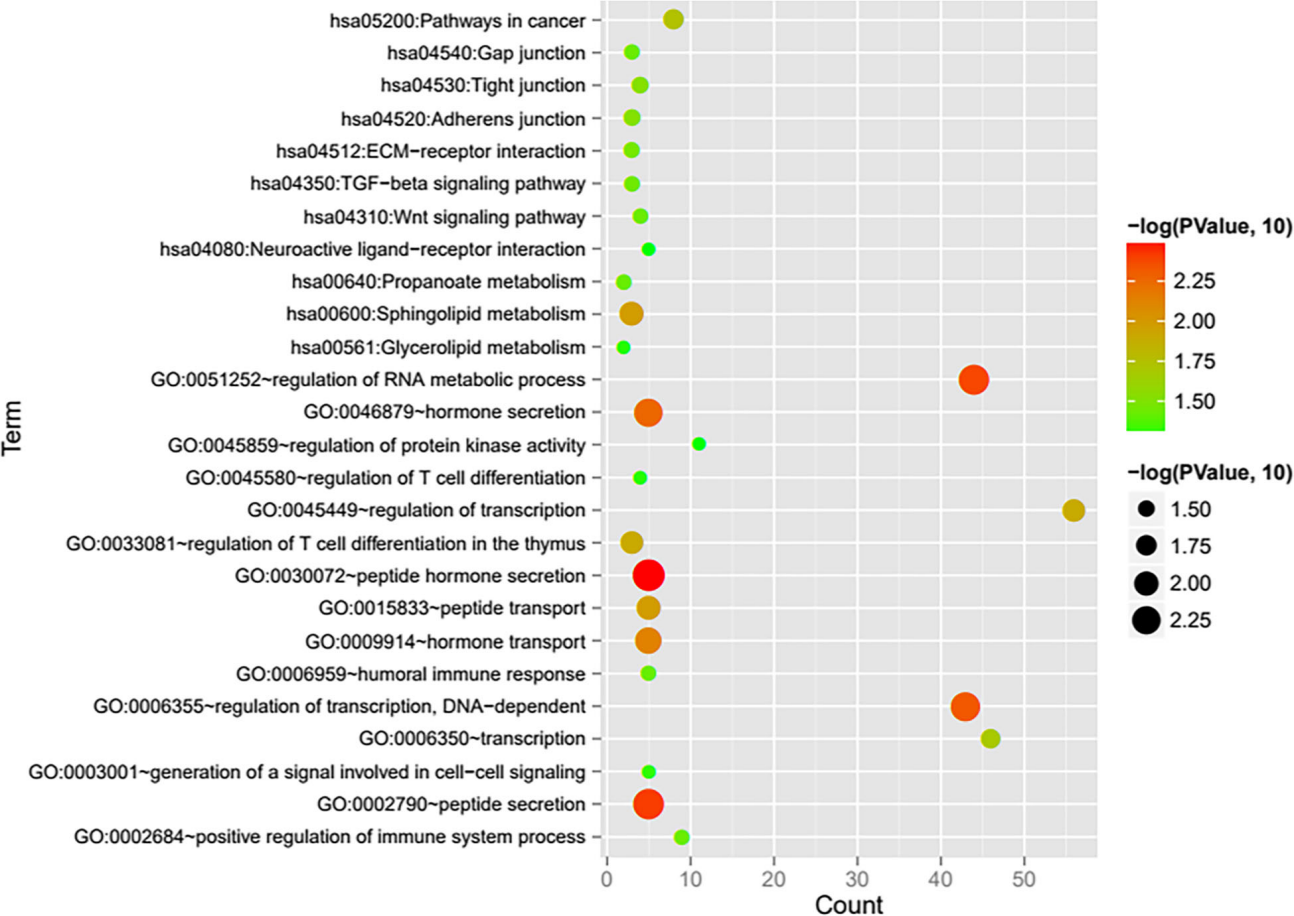
GO biological process nodes and KEGG signal point distribution maps. The horizontal axis represents the number of genes, and the vertical axis represents the name of the item. The color and size of the dots represent significance. The larger the dots, the closer the color is to red, the higher the significance

First of all, the downloaded expression spectrum data set is normalized. The normalized expression values are shown in Supplementary Table 2. The box diagrams before and after normalization are shown in Supplementary Figure 1. After re-annotation by detecting platform information, a total of 289 lncRNAs and 18197 mRNAs were obtained (the annotation information is also shown in the “type” column in Supplementary Table 2). The distribution density curves of lncRNAs and mRNAs after normalization are shown in Supplementary Figure 2. According to the sample disease information, the sample was divided into 12 SCI-pain and 13 SCI-no-pain CTRL control sample groups, and then 338 significant differentially expressed RNAs satisfying the threshold requirement were obtained by Limma co-screening, of which 187 were significantly downregulated (SCI-pain) and 151 significantly upregulated (SCI-no-pain) expression. The significant differentially expressed RNA-log_2_FC-log_10_ (FDR) volcano map is shown in Fig. 1a. Green dots indicate significant differentially expressed RNAs, red horizontal dashed lines indicate FDR < 0.05, and two red vertical lines indicate |Log2FC| > 0.5. A list of significant differentially expressed RNAs is shown in Supplementary Table 3. A bidirectional hierarchical clustering heat map based on the significant differentially expressed of RNA expression levels obtained by screening is shown in Fig. 1b. It can be seen from the figure that the RNA expression values obtained by screening can separate different types of samples and have clear colors, indicating that the RNAs screened in the pain and no-pain control groups are characteristic of the samples. Then, the screened mRNAs in significant differentially expressed RNAs were then subjected to DAVID-based GO biological processes and KEGG signaling pathway enrichment analysis annotations. Fifteen significantly related GO biological processes and 11 KEGG signaling pathways were obtained, as shown in Table 2, and the visualization is shown in Fig. 2. GO biological process nodes and KEGG signal point distribution maps that are significantly associated with mRNA in significant differentially expressed RNAs. The horizontal axis represents the number of genes, and the vertical axis represents the name of the item. The color and size of the dots represent significance. The larger the dots, the closer the color is to red, the higher the significance. The results showed that significant differentially expressed mRNAs were significantly associated with biological processes such as hormone secretion and transport, and were significantly involved in KEGG signaling pathways such as ECM-receptor interaction and TGF-beta signaling.

**Table 2:** Annotations of GO biological process nodes and KEGG signaling pathways that are significantly associated with mRNA in significant differentially expressed RNAs

Category	Term	Count	***P*** value
Biology process	GO:0030072 ~ peptide hormone secretion	5	3.301E−03
	GO:0002790 ~ peptide secretion	5	3.965E−03
	GO:0051252 ~ regulation of RNA metabolic process	44	4.240E−03
	GO:0006355 ~ regulation of transcription, DNA-dependent	43	4.838E−03
	GO:0046879 ~ hormone secretion	5	5.553E−03
	GO:0009914 ~ hormone transport	5	7.518E−03
	GO:0015833 ~ peptide transport	5	1.055E−02
	GO:0033081 ~ regulation of T cell differentiation in the thymus	3	1.258E−02
	GO:0045449 ~ regulation of transcription	56	1.279E−02
	GO:0006350 ~ transcription	46	2.036E−02
	GO:0002684 ~ positive regulation of immune system process	9	3.657E−02
	GO:0006959 ~ humoral immune response	5	3.700E−02
	GO:0003001 ~ generation of a signal involved in cell-cell signaling	5	4.639E−02
	GO:0045580 ~ regulation of T cell differentiation	4	4.723E−02
	GO:0045859 ~ regulation of protein kinase activity	11	4.908E−02
KEGG pathway	hsa00600:Sphingolipid metabolism	3	1.048E−02
	hsa05200:Pathways in cancer	8	1.803E−02
	hsa04530:Tight junction	4	2.949E−02
	hsa04520:Adherens junction	3	2.978E−02
	hsa04512:ECM-receptor interaction	3	3.345E−02
	hsa04350:TGF-beta signaling pathway	3	3.501E−02
	hsa04540:Gap junction	3	3.604E−02
	hsa04310:Wnt signaling pathway	4	3.614E−02
	hsa00640:Propanoate metabolism	2	3.673E−02
	hsa00561:Glycerolipid metabolism	2	4.751E−02
	hsa04080:Neuroactive ligand-receptor interaction	5	4.943E−02

### WGCNA algorithm screens for modules and RNAs that are significantly associated with disease characterization

**Fig. 3:**
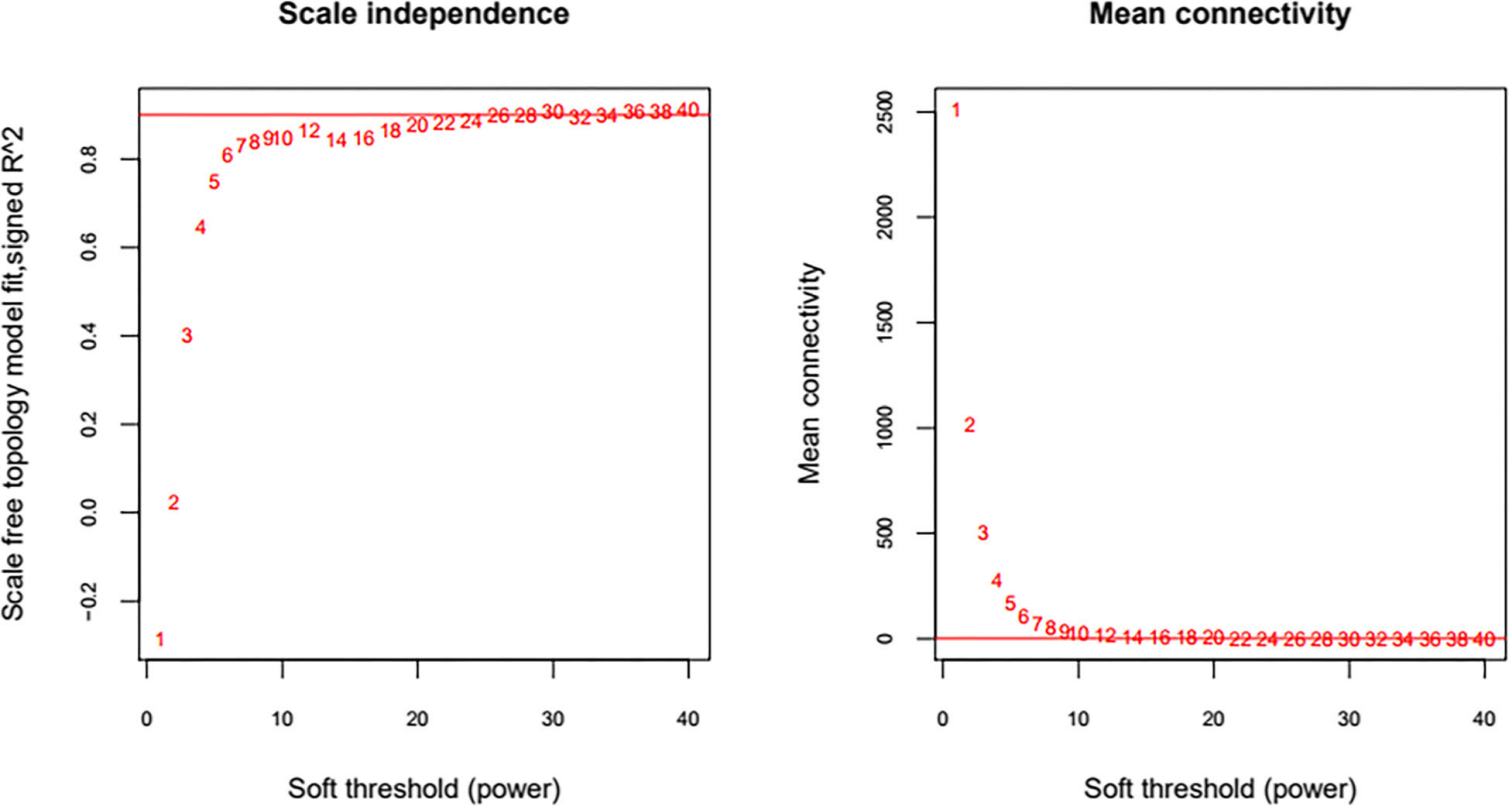
Left: Adjacency matrix weight parameter power selection graph. The horizontal axis represents the weight parameter power, and the vertical axis represents the square of the log(k) and log(p(k)) correlation coefficients in the corresponding network. The red line indicates the normalized line where the square of the correlation coefficient reaches 0.9. Right: Schematic diagram of average connectivity of RNA under different power parameters. The red line indicates the value of the average connectivity of the network node (1) under the value of the power parameter of the adjacency matrix weight parameter on the left

**Fig. 4:**
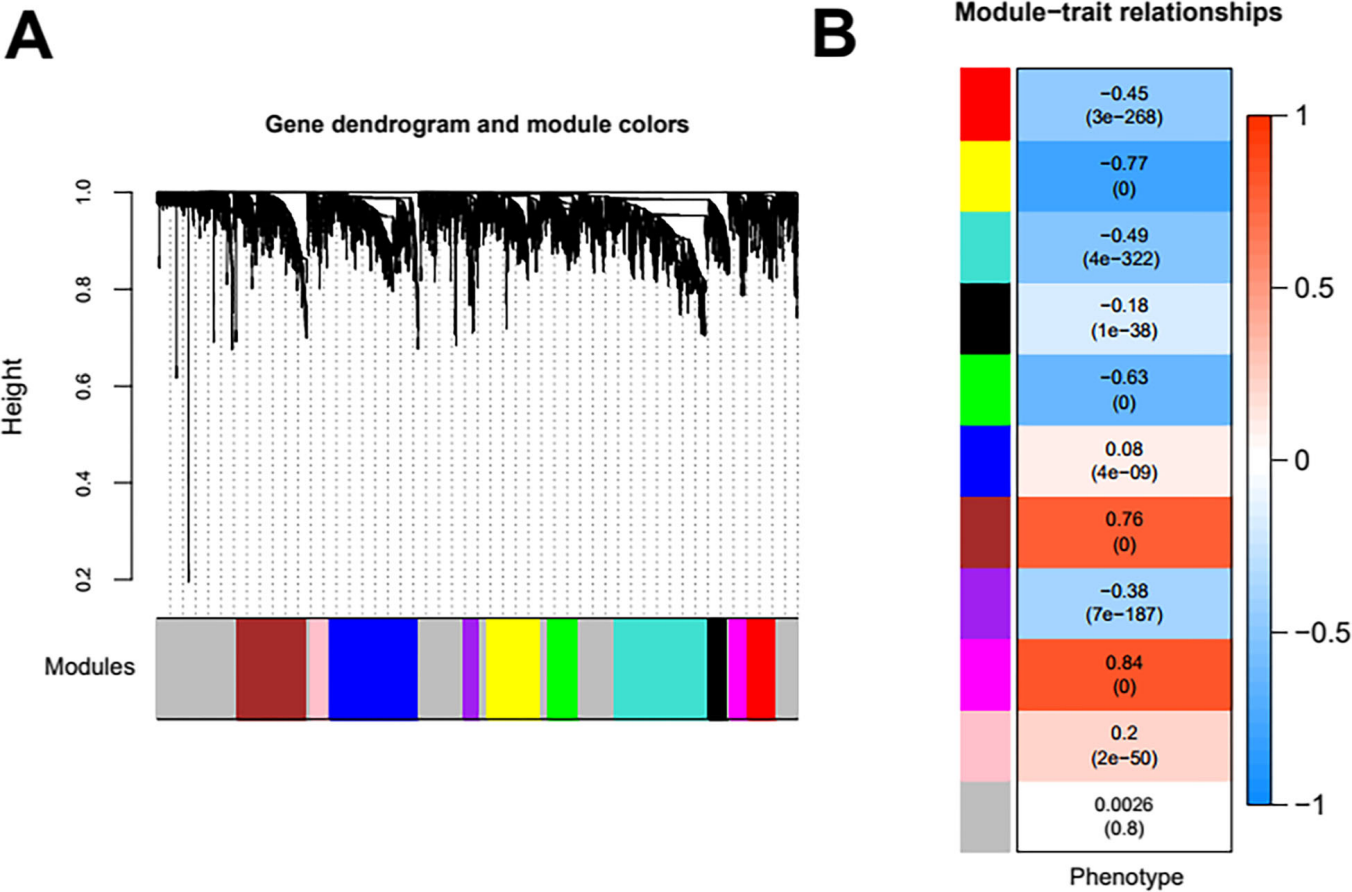
**a** Module partitioning tree diagram. Each color represents a different module. **b** Heat map of the relationship between different color modules and disease characterization. The color from blue to orange indicates a negative to positive change in the relationship with the disease

All RNAs detected by E-GEOD-69901 were subjected to analysis and screening based on WGCNA algorithm. In order to satisfy the preconditions of scale-free network distribution as much as possible, we need to explore the value of power of the adjacency matrix weight parameter: set the network construction parameter selection range and calculate the scale-free topology matrix. As shown in Fig. 3, we select the value of power when the squared value of the correlation coefficient reaches 0.9 for the first time, that is, power = 30. The average node connectivity of the co-expression network constructed at this time is 1, which is completely consistent with the nature of small-world networks. Then calculate the coefficient of dissimilarity between gene points, and obtain a systematic clustering tree. The minimum number of genes for each RNA module is 100, and the pruning height is cutHeight = 0.99. Through Fig. 3, we get the module division tree diagram of Fig. 4a. Each color represents a different module. A total of 10 modules other than gray were obtained by screening; then the correlation between the modules obtained by each division and the disease characterization was calculated. Figure 4b shows that the heat map of the relationship between different color modules and disease characterization. The color from blue to orange indicates a negative to positive change in the relationship with the disease. The results show that a total of 3 modules exhibit a very significant positive correlation with disease characterization: brown, magenta, and pink.

**Fig. 5:**
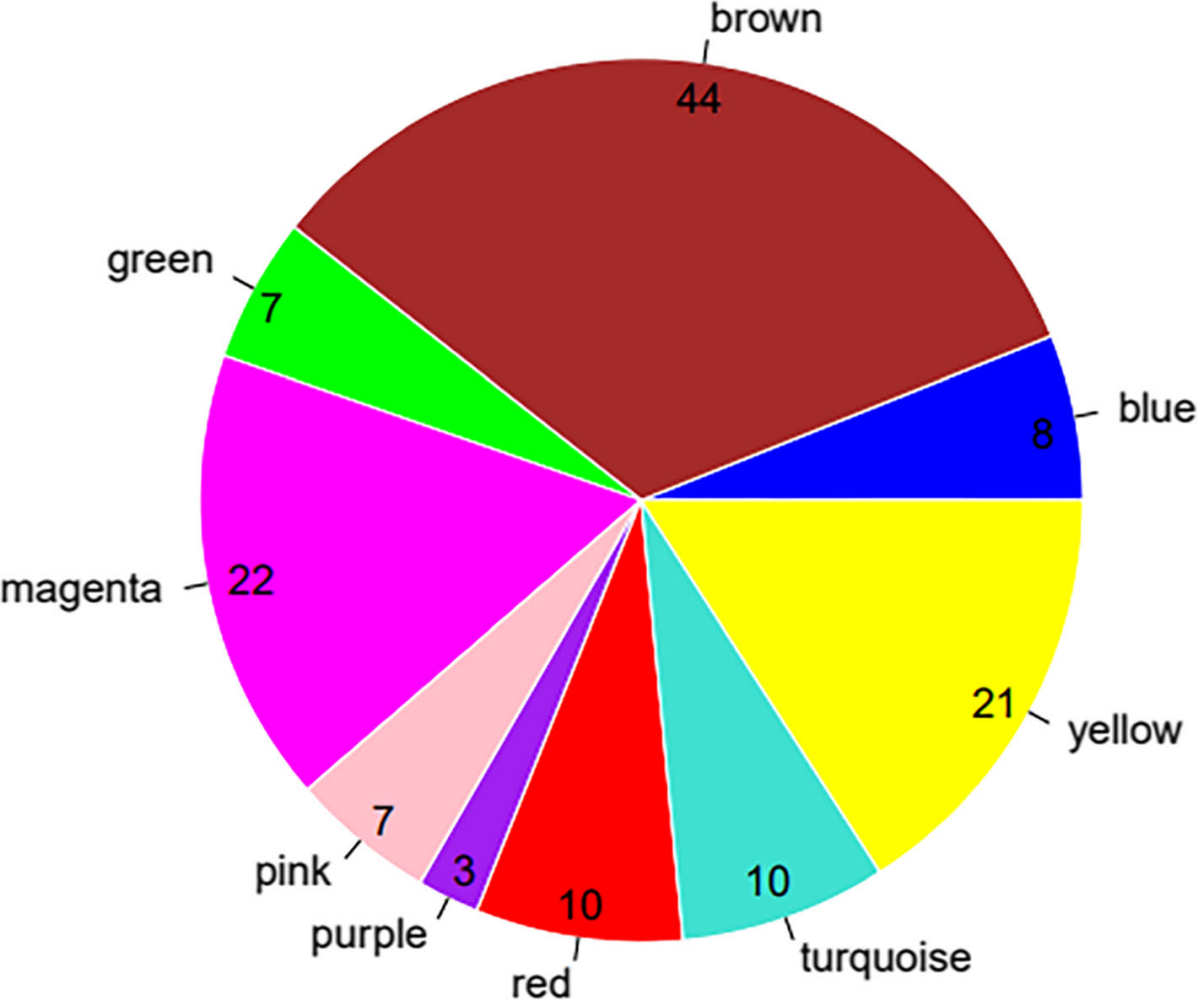
Significant differentially expressed RNAs in the module distribution ratio pie chart (except the gray module).

After the calculation according to the hypergeometric enrichment algorithm described in the method, the significant differentially expressed RNAs screened in the previous step are mapped into each module. Figure 5 shows the significant differentially expressed RNAs in the module distribution ratio pie chart (except the gray module). A total of 333 significant differentially expressed RNAs were distributed in each module (except the gray module), and the number distribution was as shown in Fig. 5. Stabilization modules with significantly enriched distribution of differentially expressed RNAs were selected and the results are shown in Table 3. The results showed that the differentially expressed RNAs were significantly enriched in the brown and magenta modules, and combined with the correlation between the previous modules and disease characterization; we used the differentially expressed RNAs in the two modules of brown and magenta as subjects for further analysis. The brown module contains 44 RNAs (2 lncRNA and 42 mRNA), and the magenta module contains 22 RNAs (22 are mRNA). The distribution of significant differentially expressed RNAs in each module is shown in Supplementary Table 4.

**Table 3:** Module information statistics

Color	#RNA	Correlation	***P***_**corr**_	#DE RNAs	Enrichment fold [95% CI]	***P***_**hyper**_
Black	166	−0.176	1.15E−38			
Blue	747	0.0802	3.66E−09	8	0.174 [0.0740-0.348]	1.78E−10
**Brown**	**588**	**0.764**	**0**	**44**	**1.213 [0.855-1.686]**	**2.48E**−**01**
Green	265	−0.631	0	7	0.428 [0.169-0.904]	2.16E−02
Gray	1704	0.00259	0.849	201	1.912 [1.583-2.304]	1.59E−11
**Magenta**	**149**	**0.836**	**0**	**22**	**2.393 [1.436-3.820]**	**7.90E**−**04**
Pink	155	0.201	1.66E−50	7	0.732 [0.287-1.562]	6.06E−01
Purple	135	−0.382	6.92E−187	3	0.360 [0.073-1.084]	9.10E−02
Red	242	−0.451	3.31E−268	10	0.669 [0.314-1.269]	2.67E−01
Turquoise	796	−0.489	4.35E−322	10	0.204 [0.096-0.381]	3.74E−10
Yellow	450	−0.771	0	21	0.756 [0.457-1.190]	2.56E−01

### Co-expression network construction

**Fig. 6:**
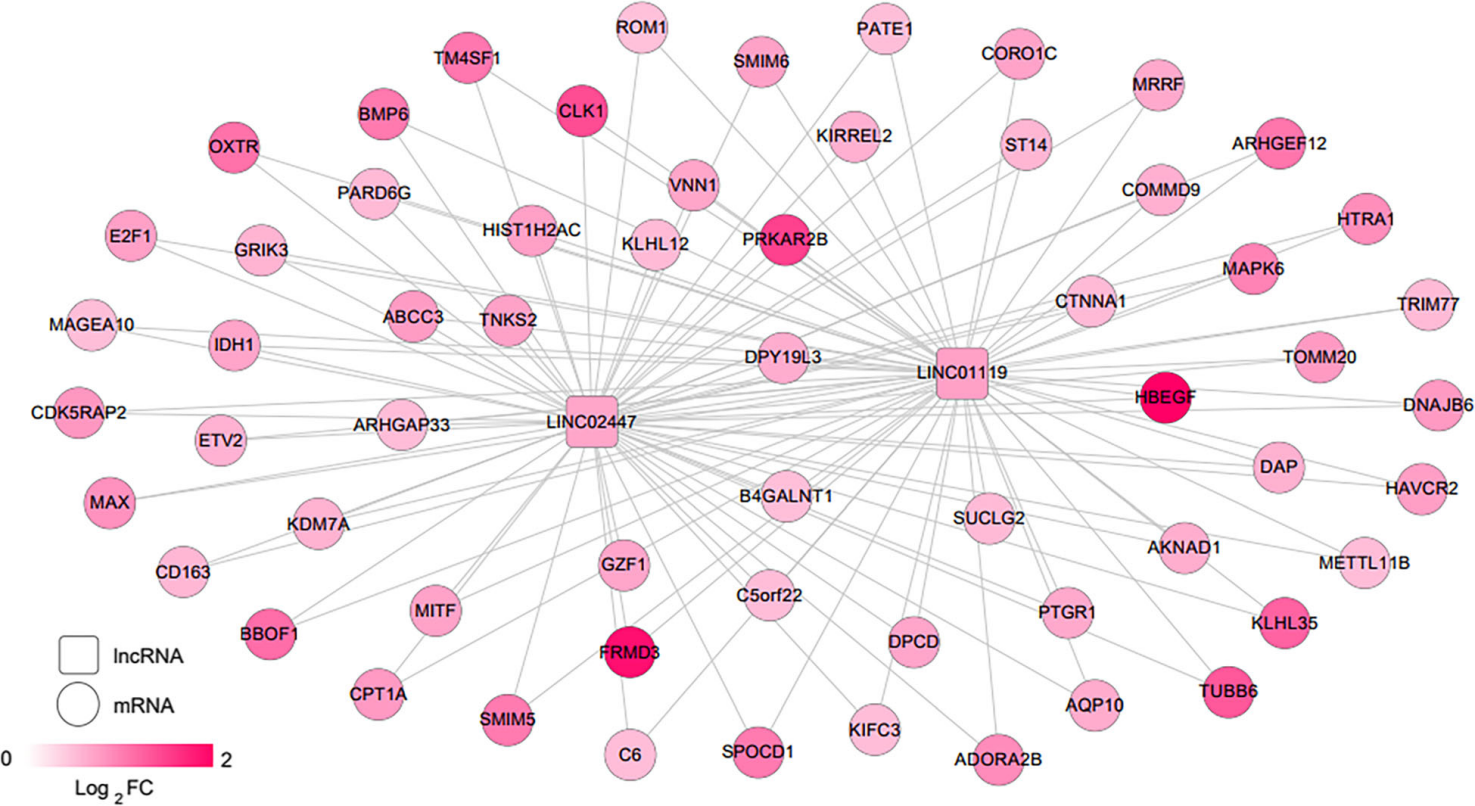
LncRNA-mRNA co-expressing genes. Square and circle represent lncRNA and mRNA. The change in node color from light to dark red indicates the change from low to high of logFC

The expression-related PCC between lncRNA and mRNA in the two target modules screened in 3.2 was calculated. A co-expression network of lncRNA-mRNA was constructed by retaining a ligation pair with a PCC above 0.4 (Supplementary Table 5), as shown in Fig. 6. The network contains 117 edges and 63 nodes, of which 2 lncRNA and 61 mRNA, both of which express significantly upregulated expression of RNAs in pain tissue. An enriched annotation analysis of the KEGG signaling pathway is then performed on the mRNAs that make up the co-expression network. Six KEGG signaling pathways were screened, as shown in Table 4. The results show that genes in the co-expression network are significantly involved in biological processes such as injury response, defense response, immune response, and inflammatory response. At the same time, these genes are significantly involved in the KEGG signaling pathways of complement and coagulation cascades, cell adhesion molecules, and ECM-receptor interactions.

**Table 4:** KEGG signaling pathways that are significantly associated with mRNAs in the co-expression network

Term	Count	***P*** value	Genes
hsa00020:Citrate cycle (TCA cycle)	2	1.15E−02	SUCLG2, IDH1
hsa05200:Pathways in cancer	4	1.35E−02	E2F1, MAX, MITF, CTNNA1
hsa04080:Neuroactive ligand-receptor interaction	3	2.67E−02	ADORA2B, GRIK3, OXTR
hsa04270:Vascular smooth muscle contraction	2	3.60E−02	ADORA2B, ARHGEF12
hsa04530:Tight junction	2	4.14E−02	PARD6G, CTNNA1
hsa04020:Calcium signaling pathway	2	4.51E−02	ADORA2B, OXTR

### Construction of SCI pain-related pathway network

**Fig. 7:**
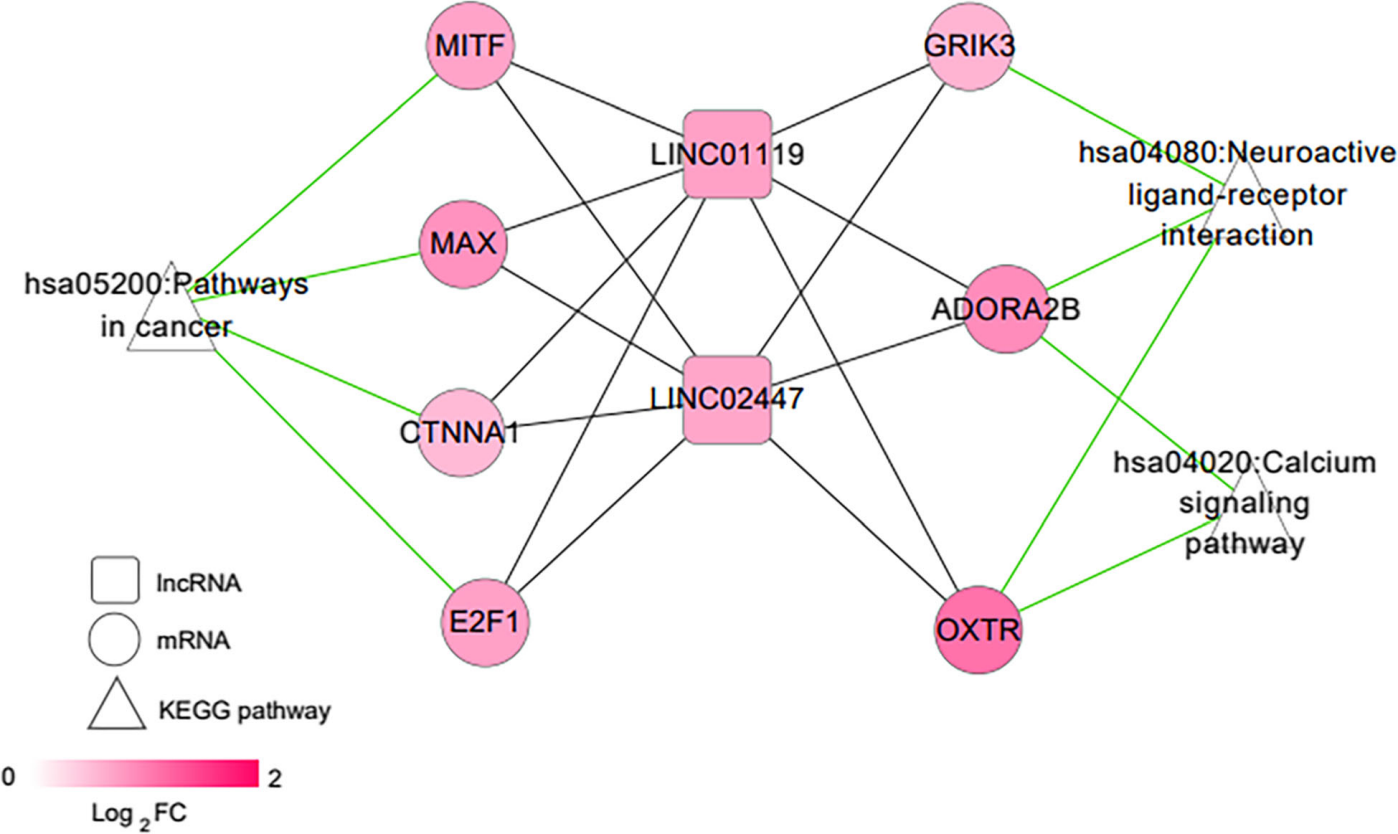
Pain-related network. Squares, circles, and triangles represent lncRNA, mRNA, and pain-related pathways. Changes in node color from light to dark red indicate a low-to-high change in logFC. The solid black line indicates the co-expression relationship between lncRNA and mRNA, and the solid green line indicates that the gene is involved in pathway junction

In the CTD database, “neuropathic pain” was used as a keyword to search for the KEGG pathway directly related to neuropathic pain. A total of 84 KEGG pathways as listed in Supplementary Table 6 was obtained. After comparing with the KEGG signaling pathway involved in the significant expression of mRNA in the co-expression network in the previous step, 3 overlapping pathways were obtained: hsa04020: calcium signaling pathway, hsa04080: neuroactive ligand-receptor interaction, and hsa05200: pathways in cancer. Based on the genes participating in the overlapping pathway, the part involved in the pathway gene is extracted from the co-expression network. Combining lncRNA-gene-pathway linkages, a pain-related network of collaterals was constructed, as shown in Fig. 7. Therefore, we speculate that the 7 genes directly involved in the pain pathway: E2F1, MAX, MITF, CTNNA1, ADORA2B, GRIK3, and OXTR are closely related to NP after SCI. In addition, LINC01119 and LINC02447 have a positive correlation with these genes at the expression level and are co-located in a module that is significantly positively correlated with disease characterization in the WGCNA algorithm results. Therefore, LINC01119 and LINC02447 may be bio-molecules that are closely related to NP after SCI.

### Real-time PCR experiment results

**Fig. 8:**
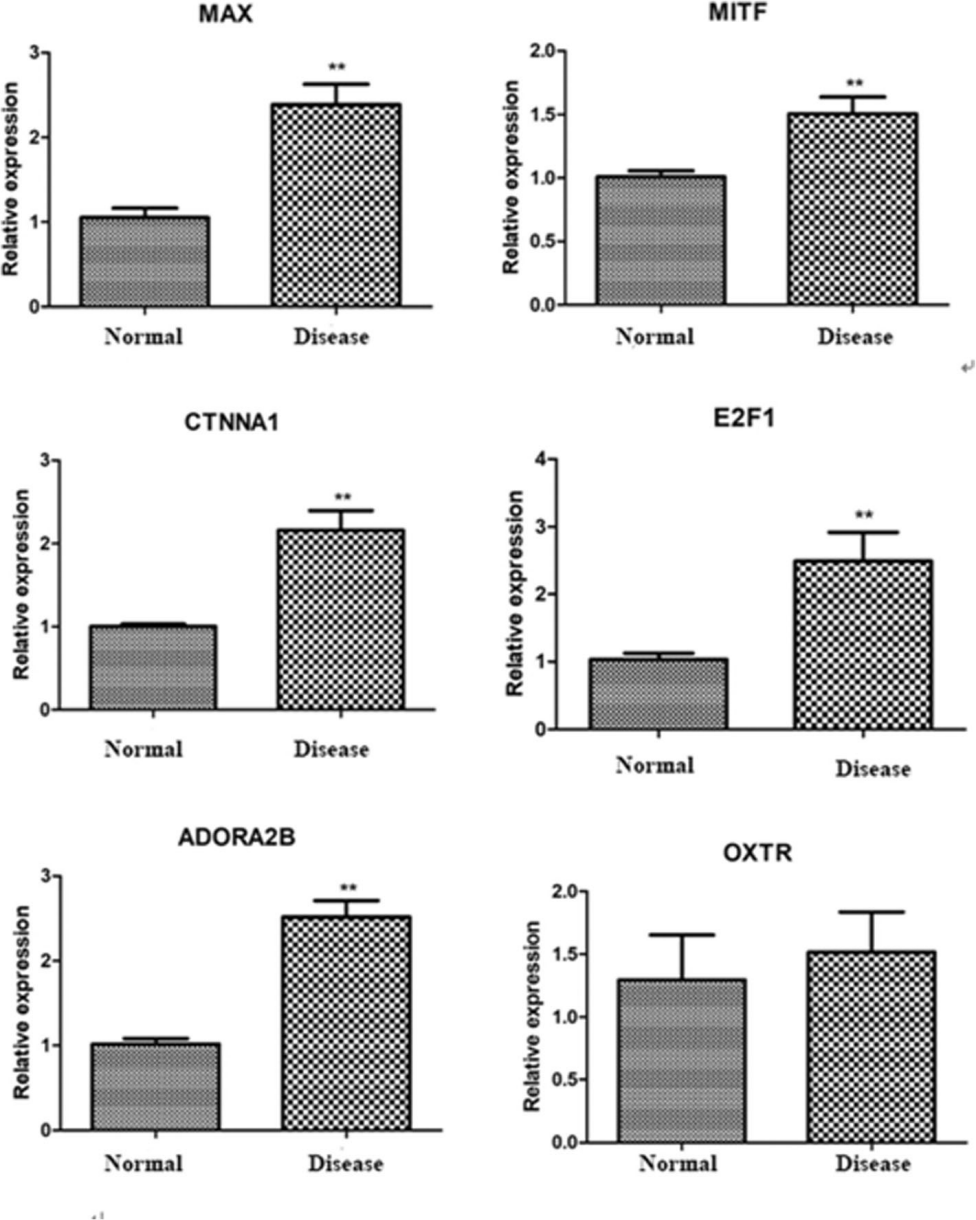
Fluorescence quantitative real-time PCR detection results. Asterisks indicate a significant difference compared with the normal group (*p* < 0.01)

Real-time PCR results showed that, compared with the normal group, the expression levels of E2F1, MAX, MITF, CTNNA1, and ADORA2B in the disease group were all significantly upregulated (*p* < 0.01). Compared with the normal group, the expression of OXTR was upregulated. There are many samples in the GRIK3 test that failed to detect the CT value. It may be that the expression abundance is too low, which makes it impossible to detect (Fig. 8).

## Discussion

The spinal cord is a vulnerable part of the central nervous system. At present, it is difficult to have effective treatment methods. The patients with spinal cord injury lose their labor and self-care ability, and cause many serious complications. Pathological neuropathic pain is more common after spinal cord injury, and there is currently no effective and effective treatment [2]. At present, the research on neuropathic pain related to spinal cord injury is mostly concentrated in mice, and research reports on human whole blood are rare [27, 28].

Based on the molecular expression level, this paper compares lncRNA and mRNA expression spectrum in peripheral blood tissue samples of NP patients after SCI, six KEGG signaling pathways were screened in the co-expression network, and three KEGG pathways with direct neuropathic pain were identified. We found that there are seven genes directly involved in the pain pathway.

Tumor-related studies have shown that the upregulation of factor E2F1 can cause pathological pain. The paracrine factors interacting with their receptors could cause the activation of downstream transcription factors such as E2F1 to upregulated expression of genes associated with pain [29]. Studies have shown that factor MITF can promote and survive osteoclast precursors, greatly enhancing the incidence of bone metastasis pain [30]. It was found in key genes related to diabetic nephropathy that CTNNA1 factor can cause other arrhythmogenic right ventricular cardiomyopathy [31]. When studied the updated mechanisms underlying sickle cell disease-associated pain, it was discovered that ADORA2B factor can be used as a target gene to cause pain. Studies have shown that mutations in the oxytocin receptor gene (OXTR) are associated with behavioral and neurological transference accuracy [32].

In summary, our results showed that the expression levels of E2F1, MAX, MITF, CTNNA1, and ADORA2B in the disease group were all significantly upregulated. Compared with the normal group, the expression of OXTR was upregulated. The data from the present study suggested that these genes may play the important role in NP (after SCI) and serve as the potential biomarkers of severe NP (after SCI) clinical diagnosis.

## Conclusion

In summary, based on the molecular expression level, this paper compares lncRNA and mRNA expressions respectively in peripheral blood samples of the patients after SCI with NP and without NP, screens GO annotations and significantly involved KEGG pathways that are significantly associated with significantly different mRNAs, and screens them with WGCNA algorithm. Significantly related modules and RNAs were constructed and co-expression networks were constructed to screen for expression-specific disease-related biomarkers related to NP after SCI in peripheral blood samples of patients. Through these disease-related molecules, we can have a better prognosis for HP after SCI. The screening of these molecules will provide an important basis for future clinical targeted research.

## Supplementary Information

**Additional file 1:.** Supplementary Figure 1. The box diagrams before and after normalization.

**Additional file 2:.** Supplementary Figure 2. The distribution density curves of lncRNAs and mRNAs after normalization.

**Additional file 3:.** Supplementary Table 1.Patient sample details. Supplementary Table 2. The normalized expression values. Supplementary Table 3. A list of significant differentially expressed RNAs. Supplementary Table 4. The distribution of significant differentially expressed RNAs in each module. Supplementary Table 5. A co-expression network of lncRNA-mRNA was constructed by retaining a ligation pair with a PCC above 0.4. Supplementary Table 6. In the CTD database, ‘neuropathic pain’ was used as a keyword to search for the KEGG pathway directly related to neuropathic pain. A total of 84 KEGG pathways as listed.

## Data Availability

The data and materials are available under the permission of the authors.

## Abbreviations

NP: Neuropathic pain
SCI: Spinal cord injury
EBI: European Bioinformatics Institute
HGNC: HUGO Gene Nomenclature Committee
WGCNA: Weighed gene co-expression network analysis
PCC: Pearson correlation coefficient
DERs: Differentially expressed RNAs
DAVID: Database for Annotation, Visualization and Integrated Discovery
GO: Gene Ontology
KEGG: Kyoto Encyclopedia of Genes and Genomes
