# Supplementary figures and images for: Screening of disease-related biomarkers related to neuropathic pain (NP) after spinal cord injury (SCI)
Source: Hum Genomics. 2021 Jan 25;15:5. doi: 10.1186/s40246-021-00303-w (PMC7831171; doi:10.1186/s40246-021-00303-w)

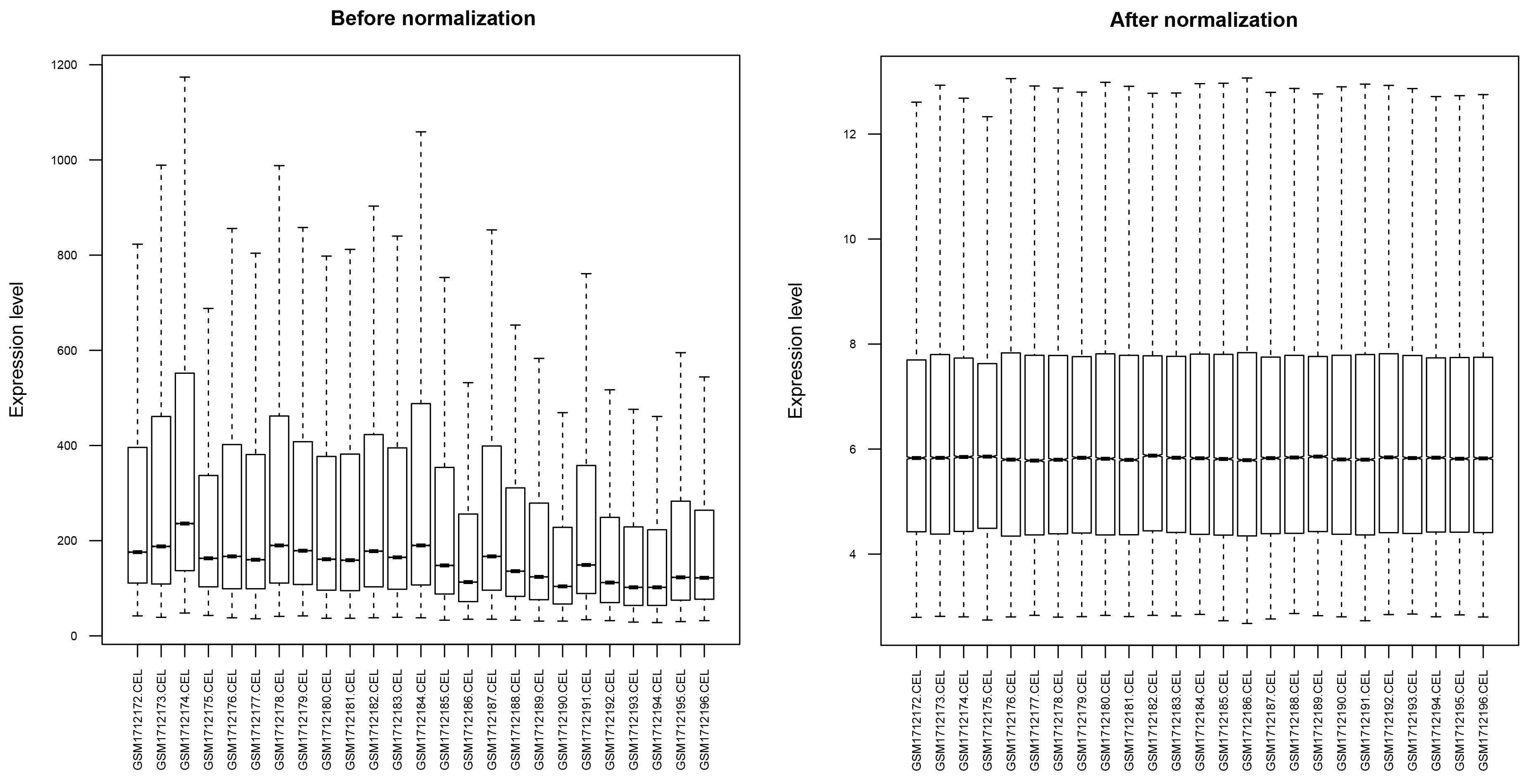

Supplement: Supplementary file 1 — Additional file 1:. Supplementary Figure 1. The box diagrams before and after normalization. [file 40246_2021_303_MOESM1_ESM.tif]

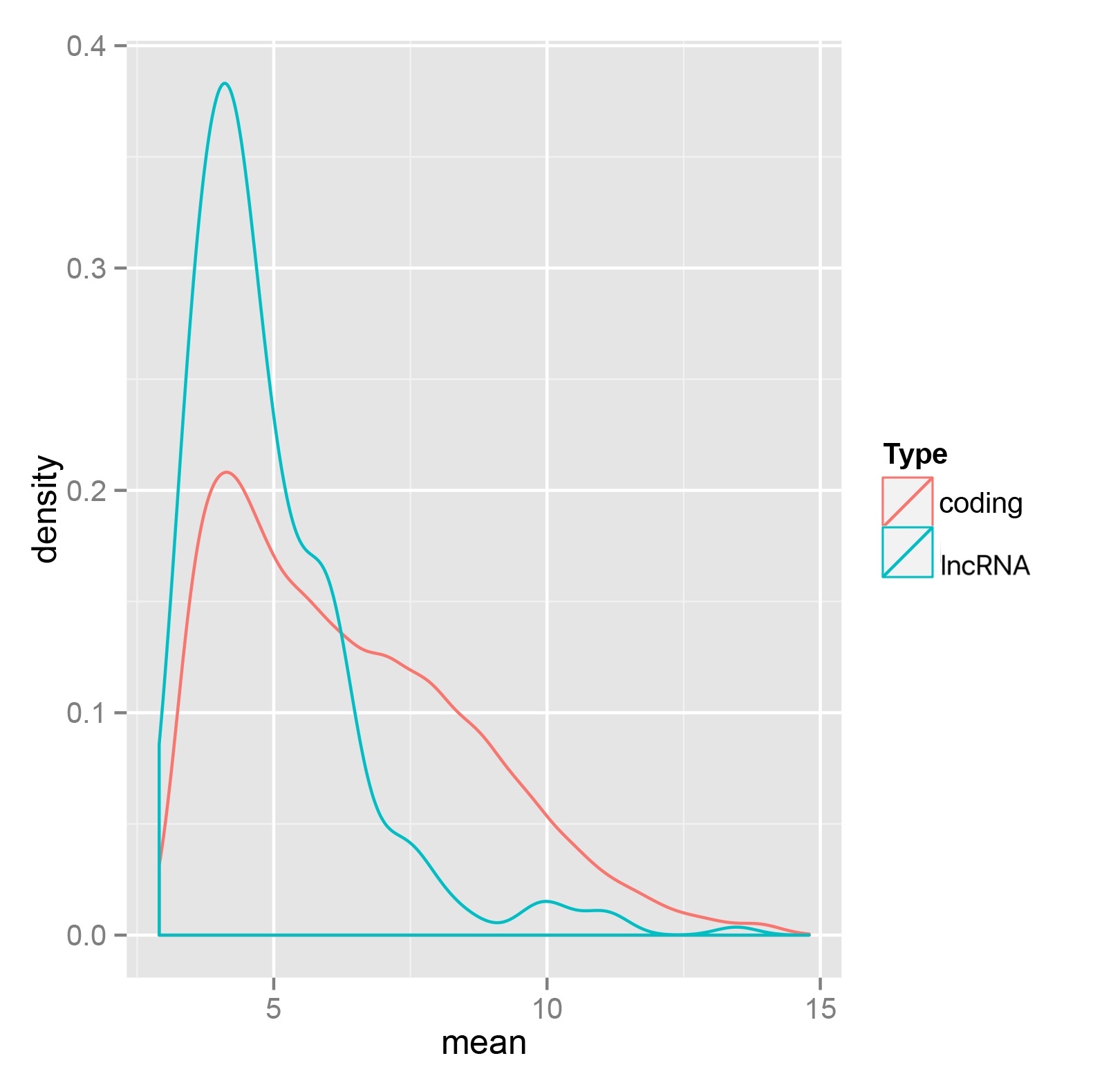

Supplement: Supplementary file 2 — Additional file 2:. Supplementary Figure 2. The distribution density curves of lncRNAs and mRNAs after normalization. [file 40246_2021_303_MOESM2_ESM.tif]
